# The Netrin-4/ Neogenin-1 axis promotes neuroblastoma cell survival and migration

**DOI:** 10.18632/oncotarget.14213

**Published:** 2016-12-25

**Authors:** Andrea A. Villanueva, Paulina Falcón, Natalie Espinoza, Solano R. Luis, Luis A. Milla, Esther Hernandez-SanMiguel, Vicente A. Torres, Pilar Sanchez-Gomez, Verónica Palma

**Affiliations:** ^1^ Laboratory of Stem Cells and Developmental Biology, Faculty of Sciences, Universidad de Chile, Santiago, Chile; ^2^ Neurooncology Unit, Chronic Disease Program, ISCIII, Madrid, Spain; ^3^ Institute for Research in Dental Sciences and Advanced Center for Chronic Diseases (ACCDiS), Faculty of Dentistry, Universidad de Chile, Santiago, Chile; ^4^ Current address: School of Medicine, Universidad de Santiago, Santiago, Chile

**Keywords:** Neogenin-1, Netrin-4, cell migration, survival, metastasis

## Abstract

Neogenin-1 (NEO1) is a transmembrane receptor involved in axonal guidance, angiogenesis, neuronal cell migration and cell death, during both embryonic development and adult homeostasis. It has been described as a dependence receptor, because it promotes cell death in the absence of its ligands (Netrin and Repulsive Guidance Molecule (RGM) families) and cell survival when they are present. Although NEO1 and its ligands are involved in tumor progression, their precise role in tumor cell survival and migration remain unclear. Public databases contain extensive information regarding the expression of NEO1 and its ligands Netrin-1 (NTN1) and Netrin-4 (NTN4) in primary neuroblastoma (NB) tumors. Analysis of this data revealed that patients with high expression levels of both NEO1 and NTN4 have a poor survival rate. Accordingly, our analyses in NB cell lines with different genetic backgrounds revealed that knocking-down NEO1 reduces cell migration, whereas silencing of endogenous NTN4 induced cell death. Conversely, overexpression of NEO1 resulted in higher cell migration in the presence of NTN4, and increased apoptosis in the absence of ligand. Increased apoptosis was prevented when utilizing physiological concentrations of exogenous Netrin-4. Likewise, cell death induced after NTN4 knock-down was rescued when NEO1 was transiently silenced, thus revealing an important role for NEO1 in NB cell survival. *In vivo* analysis, using the chicken embryo chorioallantoic membrane (CAM) model, showed that NEO1 and endogenous NTN4 are involved in tumor extravasation and metastasis. Our data collectively demonstrate that endogenous NTN4/NEO1 maintain NB growth via both pro-survival and pro-migratory molecular signaling.

## INTRODUCTION

Cancer is a complex chronic disease, characterized by the uncontrolled growth and dissemination of tumor cells. Within the several varieties of cancer, pediatric solid tumors represent about 30% of pediatric cancers, including brain tumors, rhabdomyosarcoma, Wilms' tumor, osteosarcoma, and neuroblastoma (NB) [1]. In general, these tumors arise as a result of the imbalance between proliferation/apoptosis and cell differentiation during development [2]. Particularly, NB arises from neural crest cells in the symphatoadrenal lineage that develops from the dorsal root ganglion (DRG) and adrenal gland [3]. Little is known about the specific genes and signaling pathways that are involved in the development and spread of this aggressive and highly metastatic disease [3]. Hence, it is important to understand its etiology and the molecular mechanisms involved in tumor onset and progression control.

In this context, an important molecule is NEO1, which is expressed during the development of the DRG [4]. NEO1 is a member of the immunoglobulin superfamily of transmembrane protein receptors, involved in a variety of features associated with tumor progression [5] including proliferation [6], angiogenesis [7], apoptosis [8], and migration [9] in several tissues, during development and in adult homeostasis [reviewed in [10]). Both NEO1 and its homologue, the Deleted in Colorectal Cancer receptor (DCC) [10], act as dependence receptors that induce apoptosis in the absence of their ligands (dependence factors) [11]. DCC primarily signals upon binding of the Netrin family ligands [12], whereas NEO1 signals through ligands of the Repulsive Guidance Molecule (RGM) family [8]. However, recent research has proposed that NEO1 may also act via Netrin ligands, thus avoiding its pro-apoptotic activity and promoting a survival factor function [13] in certain physiological contexts as described in β-pancreatic islets [14].

The Netrin ligands belong to the superfamily of laminin type proteins, which include five distinct members including Netrin-1, -2, -3, -4, -5 and –G, where NTN1 and NTN4 are the most characterized ligands [13]. Structurally, these molecules consist of an N-terminal domain, laminin VI, a central domain, laminin V (EGF repeated V1, V2, and V3), and one positively charged C-terminal domain. NTN4 is the most distant member of this family because its primary sequence and globular structural domain are more similar to that of Laminin V than to that of other Netrins [15]. Netrins bind to their receptors, DCC/NEO1 via the Laminin type VI domain [13]. Only the C-terminal domain of the ligand has affinity for proteoglycans and thus serves to locate Netrins to the cell surface or in the extracellular matrix (ECM) [16].

NTN1 is a critical axonal guidance protein during embryonic development, cell migration and morphogenesis, and its expression has been reported in the DRG [4]. Regarding NTN4 expression, it widely encompasses the nervous system during embryonic development and it is maintained in adult individuals. Its expression is centered in the olfactory bulb, retina, cerebellar granule cells, hippocampal and cortical neurons, and in DRG neurons [15]. The former is of interest, considering the fact that NEO1 is expressed in the DRG, suggesting a possible interaction between Netrins/NEO1 in cells that give rise to NB.

The Netrin family of ligands is highly involved in a variety of processes associated with tumor progression, however their specific contribution remains controversial. Although NTN1 was initially reported to be downregulated in NB [17], a more recent clinical study revealed that NB tissues from stage-4 patients exhibited an overexpression of NTN1, conferring a selective advantage for survival of NB cells. Disrupting the expression of NTN1 further inhibited metastasis in mouse and chicken models of NB tumorigenesis [18]. Furthermore, studies *in vitro* and patient samples have demonstrated that the interaction between NEO1 and NTN1 is associated with cell migration and invasiveness in medulloblastoma, another pediatric malignancy [19].

Contrary to the description and analyses of NTN1 contribution in pediatric cancer, the expression of NTN4 has not yet been characterized. In glioblastoma, NTN4 has been proposed to depict a biphasic function: at low physiological ligand concentrations, both proliferation and cell migration increase, whereas at high concentrations, tumor cell growth is inhibited. Reduced NTN4 expression in glioblastoma cell lines induced by serum starvation significantly decreases proliferation and motility, increasing apoptosis. This is consistent with the low expression of NTN4 in glioblastoma cells compared to its expression in healthy tissue [20]. Endogenous NTN4 also induces migration and proliferation in gastric cancer cells [21]. In breast carcinoma, NTN4 expression is most commonly detected in solid tumors than in malignant pleural effusions [22]. Combined, these findings suggest a possible biological role for NTN4 in tumor metastasis.

Since both NEO1 and Netrins are expressed in the DRG neuronal progenitors, which give rise to NB, their relationship and interaction might be relevant in the oncogenic context. However, little is known about the function that NEO1 plays in NB progression, or about the autocrine expression of Netrin ligands. Here, we provide evidence about novel roles of the NTN4/NEO1 complex in NB cell migration, survival, and *in vivo* metastasis. Furthermore, our data contribute to the identification and characterization of new therapeutic targets to inhibit NB tumor growth.

## RESULTS

### Expression of NEO1 and Netrins in NB samples and cell lines

**Figure 1:**
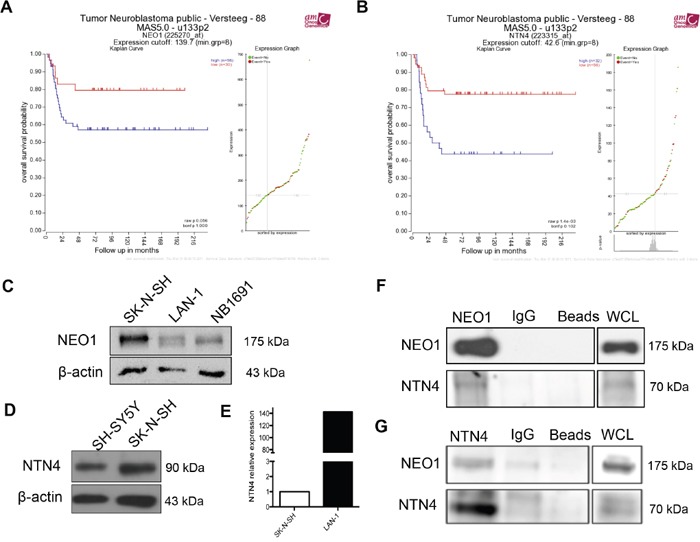
Clinical significance of NEO1 and NTN4 expression and characterization of NB cell lines **A, B**. Analysis was performed using R2 (http://r2.amc.nl) and public primary tumor NB database from 88 patients (Versteeg). The data set is separated into two categories, high and low mRNA, depending on where the values lie in relation to the median value: values above the median are high mRNA levels and those below the median, are low mRNA levels. These values are then plotted against patient survival rate in a Kaplan-Meier estimate plot. Observed is the overall survival rate according to mRNA expression of *NEO1* (A) and *NTN4* (B). **C**. Western blot against NEO1 in NB cell lines SK-N-SH, LAN-1 and NB1891. **D**. NTN4 expression in NB SH-SY5Y and SK-N-SH cell lines. Of note, NTN4 band is predicted at 69 kDa, but we detected a single band at 90 kDa, possibly due to post-translational modifications of the protein, as reported in the human Netrin-4 datasheet (R&D systems). **E**. Q-PCR analysis showing NTN4 expression in SK-N-SH and LAN-1 cells. **F, G**. Representative Western blots of protein co-immunoprecipitation assays used to evaluate interaction between NEO1 and NTN4 in SK-N-SH cells. Cells were treated for 1h with exogenous Netrin-4 (200 ng/ml) and then incubated using specific antibodies against either NEO1 (F) or NTN4 (G) followed by Western blot against NEO1 and NTN4.

In order to determine the expression of NEO1 and its ligands, NTN1 and NTN4, in primary NB tumors, and to further correlate the patient prognosis with patient survival, we reviewed public available data from R2: Genomics Analysis and Visualization Platform (http://r2.amc.nl). Specifically, we analyzed the Versteeg data set [23] that includes information from 88 patients. Our analysis depicts that high levels of *NEO1* mRNA (n=58) and *NTN4* mRNA (n=32) are associated with overall lower patient survival rates (raw p value: 0,056 and 0,0014 respectively), as seen in Figure 1A and 1B, respectively. This suggests that NEO1 and its ligand NTN4 have potential roles in NB progression. Conversely, higher *NTN1* mRNA expression (n=8) was found to be associated with lower patient survival rate (Supplementary Figure 1A).

Considering this evidence, we first sought to determine the expression of NEO1 and its ligands in two subsets of NB cell lines: SK-N-SH (*MYCN* WT), LAN-1 and NB1691 (*MYCN* amplified). As seen in Figure 1C, NEO1 is expressed in all NB cell lines studied, especially in SK-N-SH. In addition, NEO1 expression was higher in SK-N-SH, when compared to other cancer cell lines such as DAOY (medulloblastoma), U87 (glioblastoma), and HEK293 cells (Supplementary Figure 1B). Whereas NTN1 protein was barely expressed in the SK-N-SH cell line, it was detected in the two *MYCN* amplified NB (Supplementary Figure 1C). In addition, the SK-N-SH cell line did not exhibit RGMa protein expression (Supplementary Figure 1D), whereas LAN-1 did. Western Blot analysis confirmed the expression of NTN4 by the *MYCN* WT SH-SY5Y and SK-N-SH cell lines (Figure 1D). Of note, NTN4 band is predicted at 69 kDa, but we detected a single band at 90 kDa, possibly due to post-translational modifications of the protein [24]. qPCR analysis revealed that SK-N-SH and LAN-1 express *NEO1* (data not shown) and *NTN4* mRNA (Figure 1E). Next, we evaluated the putative association in a complex between NEO1 and NTN4 in SK-N-SH cells, by co-immunoprecipitation. SK-N-SH cells were incubated for 1h with exogenous Netrin-4 (200 ng/ml) and immunoprecipitation was performed with specific antibodies against NEO1 (Figure 1F) or NTN4 (Figure 1G). Our Western blot analysis shows clearly that NTN4 and NEO1 interact in SK-N-SH, favoring the hypothesis that there could be a functional relationship between these proteins in NB.

### Silencing NTN4 increases apoptosis in NB cells

Having assessed the expression and the interaction of NEO1 and NTN4 in both NB tumors and cell lines, we next decided to evaluate the potential contribution of the NEO1/NTN4 complex signaling in a variety of processes associated with tumor progression. It has been demonstrated that NTN4 is a survival factor in other cellular contexts [14] and NEO1 has been shown to behave as a death dependence factor [8]. Therefore, we sought to characterize the role of both proteins in our panel of tumor cell lines by interfering their expression. Knock-down cells for NEO1 (shNEO1) and NTN4 (shNTN4) were generated in SK-N-SH cells and in a *MYCN* amplified LAN-1 cells, using a scrambled sequence as negative control (shSCR). NTN4 expression was reduced by 70% (Supplementary Figure 2A) in SK-N-SH cells and 60% in LAN-1 cells (Supplementary Figure 2B). The expression of NEO1 was reduced by 60% in SK-N-SH (Supplementary Figure 2C) and LAN-1 cells (Supplementary Figure 2D).

**Figure 2:**
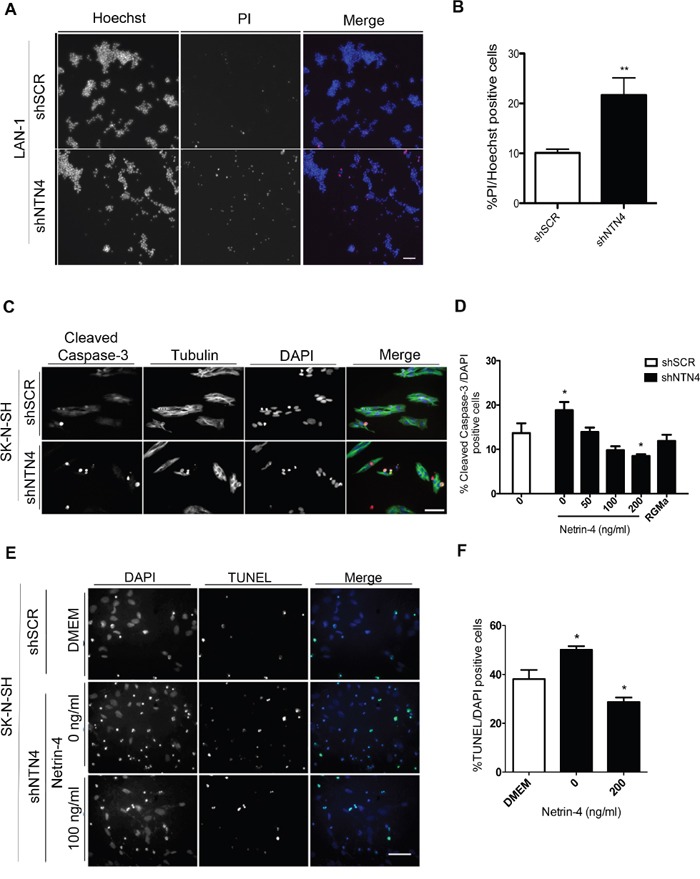
Silencing of NTN4 increases apoptosis in NB cells; exogenous Netrin-4 decreases this effect **A, B**. Representative images of propidium iodide (PI) staining in shSCR and shNTN4 LAN-1 cell cultures. Cells were serum deprived for 24 h and stained with PI and Hoechst; cell death quantification is shown in (B) *p<0,05 shSCR v/s shNTN4. Bar: 200μm. **C**. Representative images of immunofluorescence against cleaved caspase-3 in shSCR and shNTN4 SK-N-SH cells. **D**. Quantification of cell death rescue experiments in SK-N-SH cells. Cells were treated for 24 h in serum free media adding human recombinant Netrin-4 at concentrations as described. Human recombinant RGMa (100ng/ml) was used as a control. Bar:100μm **E**. Representative images of TUNEL assays made in shSCR and shNTN4 SK-N-SH cells. Cells were incubated with serum free media with or without NTN4 (200 ng/ml); quantification is shown in **F**. *p<0,05 shSCR v/s shNTN4 (0 and 200 ng/ml). Bar:100 μm.

In order to evaluate the potential role of NTN4 in cell survival, we first analyzed whether silencing endogenous NTN4 increases apoptosis in LAN-1 cells grown under serum starvation. shNTN4 LAN-1 cells were cultured without serum for 24 h and dead cells were analyzed using propidium iodide staining, while Hoechst was used to stain total nuclei (Figure 2A). We observed an increment of cell death in shNTN4 LAN-1 cells, when compared to shSCR control (Figure 2B). Next, we analyzed whether silencing endogenous NTN4 also increases apoptosis in SK-N-SH cells grown under serum starvation, and if so, whether this could be reverted with exogenous Netrin-4. As NEO1 mediates apoptosis signals through caspase-3 [25], we conducted immunofluorescence on shSCR and shNTN4 SK-N-SH cells labeled with an antibody against the cleaved caspase-3 epitope (Figure 2C). Quantification of the fluorescence demonstrates that shNTN4 cells have a greater cleaved-caspase-3 signal compared to that of the shSCR cells under serum-starvation. This signal was already reduced in the presence of minimal exogenous concentration of Netrin-4 (50ng/mL), and was further diminished with higher Netrin-4 concentrations (Figure 2D). Recombinant RGMa (100ng/ml) was used as a positive control of NEO1-mediated apoptosis avoidance [7, 8]. A TUNEL assay, conducted under serum deprivation, corroborated that shNTN4 SK-N-SH cells showed an increase of TUNEL positive cells compared with the control shSCR cells, depicting higher apoptosis in shNTN4 cells (Figure 2E). shNTN4 cells treated with exogenous Netrin-4 exhibited reduced apoptosis (Figure 2F). Since knocking down the ligand resulted in increased apoptosis, we conclude that endogenous NTN4 behaves as a survival factor in NEO1-expressing NB cells.

Interestingly, we also noticed a diminished proliferation after silencing NTN4 in SK-N-SH cells (Supplementary Figure 3A-3C), although this could be the result of increased cell death in shNTN4 cells.

### Apoptosis induced by NTN4 silencing is reversed upon NEO1 knock-down

**Figure 3:**
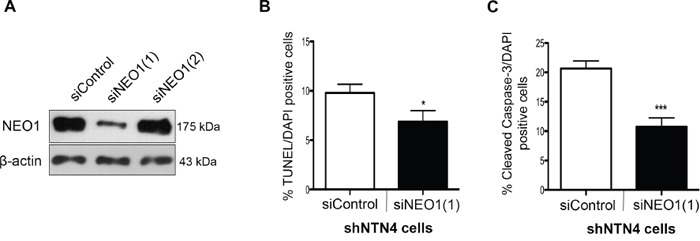
NEO1 knock-down reverses apoptosis induced by NTN4 silencing in SK-N-SH cells **A**. Two siRNA sequences were tested to silence NEO1 in NTN4 knock-down cells. Western blot revealed efficient silencing of NEO1 with the siNEO1(1) sequence. **B**. Quantification of TUNEL assay in 48 h serum deprived shNTN4 cells transfected with siRNAs as indicated. *p<0,05 siControl v/s siNEO1(1). **C**. Quantification of immunofluorescence against cleaved Caspase-3 made in 48 h serum deprived shNTN4 cells transfected with either siControl or siNEO1(1) *p<0,05 siControl v/s siNEO1(1).

NEO1 has been suggested to be a dependence receptor in specific cellular contexts driving positive cell signaling (proliferation, migration, survival), but whether or not NTN4 controls its pro-apoptotic role remains unknown. Having demonstrated that NTN4 is a survival factor in NB cells, we next analyzed whether NEO1 acts as its death dependence factor and as such, if its knock-down can revert the apoptosis induced by NTN4 silencing. For this purpose, we transfected siRNAs against NEO1 in shNTN4 SK-N-SH cells. siRNA efficiency was analyzed via Western blot and since siNEO1(1) reduced NEO1 protein levels significantly (Figure 3A), this siRNA sequence was chosen for further analysis. shNTN4 cells were transfected with siNEO1(1) and siControl and 48 h later, cells were serum starved for 48 h and fixed. Apoptosis was evaluated via TUNEL (Figure 3B) and immunofluorescence against Cleaved-Caspase-3 (Figure 3C). Both assays showed a significant decrease in apoptosis in the shNTN4 background when silencing NEO1, indicating that NTN4 is a survival factor that controls NEO1 pro-apoptotic activity.

### NEO1 and NTN4 promote *in vitro* chemotactic cell migration

**Figure 4:**
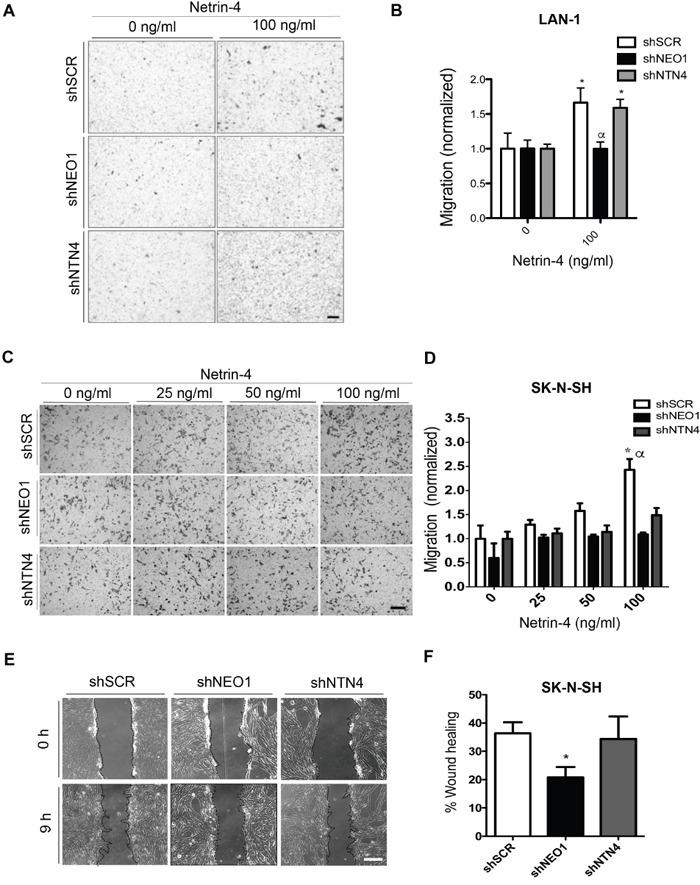
NEO1, acting through NTN4, promotes in vitro chemotactic cell migration in NB cells **A**. Representative images of transwell assay with LAN-1 knock-down cells. shSCR, shNEO1, and shNTN4 LAN-1 cells migrated for 6 hours under exogenous Netrin-4 (100 ng/ml), added to the serum free media in the botton chamber. Bar:100μm. **B**. Quantification of LAN-1 transwell assay * p<0,05 shSCR or shNTN4 0 v/s 100ng/ml NTN4, α p<0,05 shSCR or shNTN4 v/s shNEO1. **C**. Representative images of transwell assay made with SK-N-SH knock-down cells. shSCR, shNEO1, and shNTN4 SK-N-SH cells migrated for 4 hours under different concentrations of exogenous Netrin-4 present in the serum free media placed in the botton chamber. Bar:100μm. **D**. Quantification of transwell assay * p<0,05 shSCR 0 v/s 100ng/ml NTN4, α p<0,05 shSCR v/s shNEO1 or shNTN4. **E, F**. Wound healing assays of stable knocked-down SK-N-SH cells using shRNAs as indicated. Representative images (E) were quantified after 9 hours (F). Bar:100μm; *p<0,05 shSCR v/s shNEO1.

Considering that NEO1 is involved in neuronal cell migration [9], we aimed to address whether this function was relevant in the tumor context and if it was dependent of its ligand NTN4. Using transwell chemotaxis assays, we examined the function of NEO1 in sensing and guiding tumor NB cells, and analyzed whether NTN4, a known chemotactic molecule, influenced this process. Previous studies have revealed a dose-dependent effect of NTN4 in glioblastoma cell proliferation and motility [20]. Therefore, we compared the migratory behavior of shNEO1, shNTN4, and shSCR LAN-1 in the presence of 100ng/ml of Netrin-4 as a chemoattractant in the bottom chamber (representative images shown in Figure 4A). We observed that shSCR and shNTN4 LAN-1 cells migrate positively towards the chamber containing Netrin-4 (Figure 4B). Conversely, shNEO1 cells cannot migrate positively in presence of Netrin-4 (Figure 4B), therefore, these cells cannot sense Netrin-4 as a chemotactic molecule, *ergo* disabling the cell migration execution. Next, we wondered if this mechanism was conserved in *MYCN* WT cells, such as SK-N-SH cells, by using shSCR, shNEO1 and shNTN4 cells, and by increasing concentrations of exogenous Netrin-4 (25, 50, 100 ng/ml) in the bottom chamber (Figure 4C). The migration of shSCR cells augmented with increasing concentrations of NTN4 (Figure 4D). Our results depict a negative chemotaxis response due to the fact that shNEO1 cells did not sense NTN4, which is in line with the results observed for the LAN-1 NB cell line. Strikingly, both shNEO1 and shNTN4 cells migrated less than shSCR cells and their migration did not improve with increasing concentrations of Netrin-4 (Figure 4D). Even when we tested higher concentrations of Netrin-4 (200 and 500 ng/ml) as a chemoattractant stimulus for shNTN4 SK-N-SH cells, we did not observe any increases in cell migration (data not shown). Previous reports have shown that endogenous NTN4 promotes cell migration in gastric cancer cells [21]. Indeed, NTN4 is required for proper endothelial cell migration, adhesion, and focal adhesion contacts, through the binding with a_6_β_1_ integrin [26]. This suggests that NTN4 might be necessary to initiate NB cell migration, possibly acting through non-canonical receptors. The difference in shNTN4 cell migration between the two NB cell lines is probably due to the differential NTN1 expression, which is not present in the SK-N-SH cells, but is expressed in LAN-1 cells. This protein partially rescued the shNTN4 phenotype in the second cell line (Supplementary Figure 1C). Together, these results suggest that NEO1 promotes cell migration through NTN4 in NB.

We decided to complement our SK-N-SH cell migration analysis by performing a functional complementary approach, such as the wound healing assay, in presence of low serum (2,5%). shNEO1 cells migrated significantly less, when compared to shSCR and shNTN4 (Figure 4E). Quantification of the wounded area is shown in Figure 4F. The impaired wound closure in shNEO1 cells confirmed the importance of NEO1 in NB cell migration, and thus in tumor migration.

### NEO1 regulates apoptosis and cell migration through NTN4

**Figure 5:**
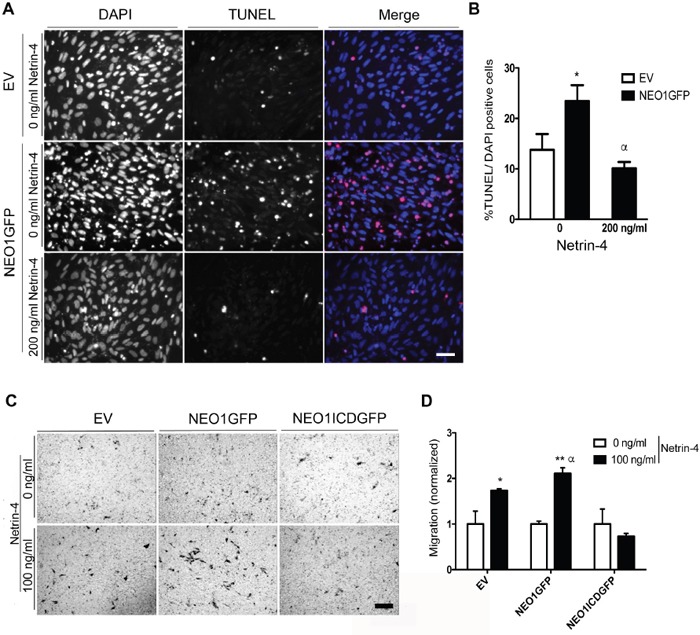
Overexpression of NEO1 induces apoptosis that can be rescued by exogenous Netrin-4 and promotes cell migration through NTN4 chemotaxis **A**. Representative images of TUNEL assay made with SK-N-SH cells transfected with NEO1 (NEO1GFP) or empty vector (EV) and treated for 24 h with serum free media with or without Netrin-4 (200 ng/ml). Bar:100 mm. TUNEL positive cells are shown in red and were quantified in **B**. *p<0,05 EV versus NEO1GFP (0 ng/ml), α *p<0,05 NEO1GFP (0n/ml) vs NEO1GFP (200ng/ml Netrin-4). **C**. Representative images of transwell assays using SK-N-SH cells that overexpress NEO1 (NEO1GFP), or its intracellular domain (NEOICDGFP). An empty vector (EV) was utilized as a control. Cells migrated for 4 hours using 100 ng/mL Netrin-4 as a chemotactic stimulus Bar: 100μm. **D**. Quantification of transwell assay shown in (C) * p<0,05, **p<0,001, α p<0,05.

Our previous results demonstrate that silencing NTN4 increases apoptosis, an effect that is reverted after transient NEO1 knock-down (Figure 3). In order to validate whether NTN4/NEO1 acts as a signaling complex in NB we analyzed the effect of NEO1 overexpression in SK-N-SH cells. Relative protein expression analysis revealed a two-fold increase in the levels of NEO1 in NEO1GFP compared to cells that were transfected with an empty vector (EV) (Supplementary Figure 2E, 2F). In agreement with our hypothesis, results indicate that overexpressing NEO1 (NEO1GFP) increased TUNEL positive cells compared to control EV cells, suggesting an increase in cell apoptosis (representative images shown in Figure 5A). Introducing exogenous Netrin-4 reduced the number of TUNEL positive cells, suggesting a reduction of apoptosis (Figure 5B). To determine whether NTN4 acts as a survival factor specifically through NEO1 or as a general survival factor, we overexpressed TrkC, a dependence receptor that acts as a tumor suppressor in NB [27] and repeated TUNEL assays. As expected, TrkC induced apoptosis in SK-N-SH cells (Supplementary Figure 3D). However, exogenous Netrin-4 did not reduce TUNEL positive cells overexpressing TrkC, suggesting that NTN4 acts as a survival factor specifically via NEO1. Taken together, these results indicate that NEO1 triggers apoptosis in the absence of NTN4, whereas the induction of cell death is inhibited if NEO1 binds to its ligand.

Finally, we decided to analyze the effect of NEO1 overexpression in the migratory assay. For that, we transfected SK-N-SH cells with either a GFP-tagged NEO1 (NEO1GFP) or the intracellular fragment of NEO1 (NEO1ICDGFP), which lacks the extracellular domain that binds to the ligands. Supplementary Figure 2E shows a scheme of the full-length NEO1 protein indicating the NEO1ICD domain localization. Transfection efficiency obtained was around 50%. We performed a transwell assay with NEO1GFP, NEO1ICDGFP, and EV cells using 100ng/mL of Netrin-4 as a chemotactic stimulus (representative images shown in Figure 5C). As shown in Figure 5D, NEO1GFP overexpressing cells migrated more than the control EV cells (α p<0,05) when the chemotactic stimulus was introduced, while NEO1ICDGFP cells did not exhibit significant migration. The lack of significant migration indicates that NEO1ICDGFP transfected cells cannot sense the NTN4 stimulus efficiently, probably due an interference of NEO1ICDGFP with the downstream signaling activities of the endogenous full length NEO1. Therefore, in NB cells, endogenous NTN4 acts as a survival factor and induces cell migration, both through NEO1 binding. Summarizing these results, we can conclude that, in NB cells, there is a delicate balance between apoptosis and cell migration controlled by the NEO1/NTN4 signaling axis.

### NEO1 and endogenous NTN4 participate in metastasis *in vivo*

**Figure 6:**
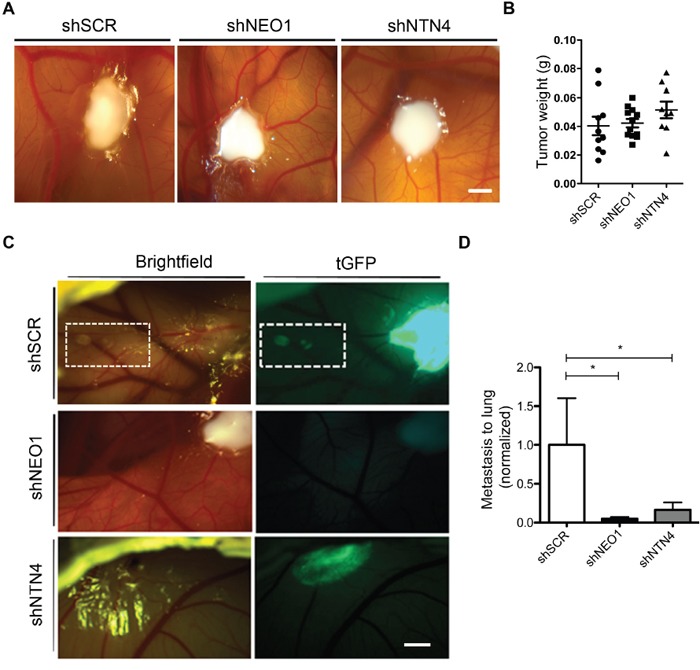
NEO1 and endogenous NTN4 participate in spontaneous metastasis in vivo CAM assays were used to evaluate spontaneous metastasis of SK-N-SH cells dropped onto CAMs of chicken embryos on day 10 of incubation (E10). Stable knock-down cells shSCR, shNEO1, and shNTN4 were used in this assay. After 7 days (E17) embryos were dissected and lung metastasis was analyzed for each cell type. **A**. Representative images of primary tumor in CAM 5 days post-dropping. Bar: 5 mm. **B**. Tumor weight from primary tumor formed by the different cell lines in CAM. **C**. On day 5 post-dropping, secondary tumors in CAM were clearly visible and were formed by shSCR cells that migrated from primary tumors. This process did not occur in the case of shNEO1 and shNTN4 cells. Bar: 5 mm **D**. Quantification of Q-PCR analysis using genomic DNA of human *Alu* sequences in lungs to evaluate metastasis for shRNA cells, compared with chicken *GAPDH* gene. *p<0,05.

After examining the potential role of endogenous NEO1 and NTN4 participating in apoptosis and cell migration, we wanted to ascertain whether they also participate in primary tumor formation and metastasis *in vivo*. We conducted a chick CAM assay and transplanted shNEO1, shSCR, or shNTN4 SK-N-SH cells, to generate primary tumors in chicken embryos (representative images are shown in Figure 6A). The resulting primary tumors were all similar, with no significant differences in tumor size and weights among the different transfected cell batches (Figure 6B). Importantly, we observed that shSCR cells formed secondary tumors in 57,2% of the embryos, characterized as GFP+ small nodules on the CAM, near the primary tumor (Figure 6C), while shNEO1 and shNTN4 transfected cells grew mostly as primary tumors (16,6 % shNEO1 and 22,2 % shNTN4; secondary tumor formation). The latter result suggests that shNEO1 and shNTN4 cells have a reduced capacity to migrate across the CAM.

Tumor metastasis involves the migration, invasion, and proliferation of tumor cells into other tissues and niches. We evaluated metastasis rate in the embryonic chicken lungs by amplifying human *Alu* sequences. The PCR results indicate that neither shNEO1 nor shNTN4 cells metastasized into the lungs (Figure 6D). Overall, these results suggest that both NEO1 and NTN4 may potentially participate in metastasis *in vivo*.

## DISCUSSION

Tumor metastasis is orchestrated by several cellular processes, such as proliferation, cell survival, apoptosis, and cell migration [28]. In this work, we show that NEO1 is involved in all of these processes, recapitulating its known role in embryonic development [10]. NEO1 [4, 29] and NTN4 [15] are expressed in sites where NB originates, such as in the DRG of the embryonic neural crest, and we hypothesize that their functions persist throughout cancer progression, as a result of an impairment in the normal developmental signaling. Clinically, NEO1 expression endures throughout NB progression [30], and often results in poor patient prognosis. Conversely, loss of DCC expression, a homolog of NEO1, correlates inversely with the degree of NB dissemination [31], acting in this context as a tumor suppressor. In line with this data, *DCC* is not expressed in SK-N-SH cells [31] or in other NB cell lines [30, 31]. This phenomenon is due to the fact that the *DCC* gene undergoes an allelic loss. In fact, reduced expression of DCC [31] has been reported in several types of cancer (prostatic, colorectal and NB), unlike NEO1, which expression is not altered [30]. The expression of NTN4, one of the described ligands of the NEO1/DCC receptor family [32, 33], is also associated with an overall poor survival rate of NB patients. Overall, these results are in agreement with our meta-analysis of repository data, confirming that both NTN4 and NEO1 expression correlates with a worse prognosis, whereas NTN1 expression behaves the opposite. In fact, previous data from other groups have shown that NTN1 loss of expression in both, patient samples and NB cell lines, might contribute to progression of NB [17].

Here, we establish that both, WT and high *MYCN* NB cell lines (represented by SK-N-SH and LAN-1), express NEO1 and NTN4. In particular, we demonstrate that both proteins interact directly in NB cell lines, conforming a signaling complex that contributes differentially in several tumoral progression processes.

### Role of NTN4 in apoptosis

Physiologically, NTN4 promotes angiogenesis [34, 24] in endothelial cells [7], driving proliferation, migration, and adhesion. Within the cancer context, NTN4 could have biphasic roles. At low physiological concentrations [24], cells exhibit high proliferation rates in glioblastoma [20] and gastric cancer [21]. At high concentrations, however, proliferation and angiogenesis are inhibited in glioblastoma [20] and colorectal cancer [35]. NTN4 could also act as a survival factor, especially because it blocks the pro-apoptotic activity of its receptors [14]. In fact, NTN4 has been shown to promote survival maintenance in beta pancreatic islets [14]. Here, by using apoptotic markers, we have confirmed that endogenous and exogenous NTN4 act as a survival factors in cells with different levels of endogenous NTN4.

### NTN4 as a survival factor through NEO1 signaling

Apoptosis increases when NEO1 is overexpressed, revealing its death dependence function [8]. This effect was reverted using exogenous Netrin-4. In addition, when NEO1 is silenced in a shNTN4 background, the apoptosis induction by the NTN4 knock-down is reverted, indicating that NTN4 is a survival factor modulating dependence receptor NEO1's pro-apoptotic downstream signaling. In addition, Netrin-4 did not reduced the apoptosis of cells overexpressing TrkC indicating that NTN4 acts as a survival factor specifically through NEO1. Thus, the NEO1/NTN4 signaling complex modulates a balance between survival and apoptosis in these cells. NTN1, another NEO1 ligand, is overexpressed in aggressive NB and is also considered a survival factor [18]. Although LAN-1 express NTN1, NTN4 acts probably as the main NEO-1-related survival factor in these cells, since cell death is triggered when NTN4 is silenced. Likewise, it has been shown in NB that NTN1 could act as a survival factor through UNC5H downstream signaling [18].

Usually, dependence receptors act as tumor suppressors [11]. However, NEO1 knock-down does not reduce cell death and its expression is maintained in several cancer cells, such as NB [30] and medulloblastoma [6]. We propose that autocrine NTN4 expression through NEO1 binding allows sustained NEO1 function in these tumors, maintaining cells in a pro-survival state.

### NEO1 acts via NTN4 in NB cell migration

In this work, we show that NEO1 is involved in NB cell migration, acting via its ligand NTN4. In other cell types, such as gastric cancer, NEO1 modulates the effect of endogenous NTN4 on motility [21]. The overexpression of NEO1 in gastric cancer increases cell motility, acquiring a migratory phenotype [36], and its knock-down reduces cell migration in the same cell types [21]. Researchers have stated these observations in other contexts, and, so far, no data have demonstrated the dose-dependent chemotactic behavior of NEO1 in NB cells.

Evaluating the effects of cell-matrix and cell-cell interactions via wound healing assays revealed that NEO1 SK-N-SH knock-down cells significantly reduce cell migration compared with shSCR (control) and shNTN4 knock-down cells. Endogenous NTN4, under these conditions, did not influence cell migration. But it is worth mentioning that our experimental conditions included low serum supplementation, which contains NTN4 as well as other Netrin ligands (data not shown). Thus, we also performed transwell assays in the absence of serum to evaluate the role and contribution of NTN4 to cell migration and the competence of NEO1 to sense NTN4 chemotactic stimulus. Knocking down NTN4 did not result in an increase in cell migration compared to the condition without a chemotactic stimulus, hence suggesting that endogenous NTN4 maintains cells in a pro-migratory state. Contrary to SK-N-SH, LAN-1 cells do express NTN1 and shNTN4 cells migrate sensing NTN4 chemoattraction; most probably NTN1 compensates the NTN4 function in these cells. Furthermore, there is evidence that demonstrates the binding of NTN4 and NTN1 to other non-canonical Netrin receptors, such as Integrins (α6β1, [26]), which might explain a NEO1-independent Netrin compensation. We observed that shNEO1 cells did not migrate more than in a condition without stimulus. These cells cannot sense the NTN4 stimulus, highlighting the importance of NEO1 in guiding cells to migrate. Overexpressing NEO1 resulted in greater cell migration compared to control cells, confirming that NEO1 has an essential role in promoting cell migration.

The dependency of NB cells to migrate through NEO1 was revealed when we overexpressed NEOICD in SK-N-SH. NEOICD cells cannot bind Netrins and, consequently, the cells did not migrate efficiently. This result also confirms the possibility that endogenous NTN4 is binding to the NEO1 receptor on the cell surface, activating its intracellular pro-migratory cascade, possibly through focal adhesion kinase (FAK) signaling. During neuronal migration and axonal guidance, NEO1 binds to FAK [37]. FAK is fundamental for focal adhesion dynamics and cell migration [38]. Therefore, NEO1 promotes cell migration, guiding the cells via the NTN4 chemotactic stimulus. This is relevant in NB cells, because the molecular mechanisms that mediate neural crest delamination are also likely to be involved in NB migration [39]. The data suggest that NEO1, expressed in DRG, is involved in the symphatoadrenal-lineage neural-crest cells migration during embryonic development. DCC is involved in the migration of neural crest cells in the formation of the bowel and pancreas [40], acting through NTN1 as a guidance molecule; as a homolog, NEO1 may also participate in this process. The role of NEO1 is probably conserved, although intracellular pathways are not shared between NEO1 and DCC. In axonal guidance for example, DCC selectively interacts with Src family kinases Fyn and Lck intracellularly [41], while NEO1 interacts additionally with SHIP1 phosphatase. The different molecules involved in each signaling pathway may explain the differences between NEO1 and DCC signaling in tumoral suppression. The evidence provided by others and the results presented here, collectively, indicate that NEO1 acts by primarily promoting tumoral migration and survival through its ligands produced by the tumoral cells [18], stroma [42], or lamina basal [7], while DCC acts mostly as a pro-apoptotic molecule [43], which expression is reduced in several tumors as NB [31] among others.

### Convergence of functions of NEO1/NTN4 in tumor progression

NEO1 and NTN4 knock-down cells generate primary tumors in CAMs, with similar weight and size compared with control tumors (shSCR). The CAM itself produces survival factors [44] and its vessels irrigate the tumors, reducing pro-apoptotic signals, which could explain the similar behavior of shSCR, shNEO1 and shNTN4 cells. As CAM cells migrate, the NEO1/NTN4 signaling complex becomes increasingly more important, deduced by the formation of secondary tumors near the primary tumors of SK-N-SH shSCR cells. shNEO1 and shNTN4 cells lacked secondary tumors, reinforcing the idea that the NEO1/NTN4 complex is biologically pivotal for cell migration process *in vivo*. Metastasis is a complex process that involves cell migration and invasion. When we evaluated the role of NEO1 in this latter process, we observed that NEO1 knock-down cells have a reduced capacity to form secondary tumors and to metastasize to lungs, revealing an impaired migration. Thus, NTN4 is also required for metastasis. This result suggests that NTN4 maintains the cells in a pro-migratory state in coordination with NEO1, activating intracellular signals. Physiologically, we hypothesize that as metastasis commences, NEO1 expressing cells sense the NTN4 gradients produced by the Netrin-producing cells, mainly located in the lamina basal of endothelia [7]. Therefore, NEO1 may have an active role in metastasis by sensing ligand gradients, promoting NTN4-guided intravasation, and colonizing new cancerous niches.

This work suggests that NEO1 acts as a tumoral progression-promoting protein, with an active role in metastasis, resembling its function in developmental cell migration. NEO1 itself induces apoptosis in certain contexts but within the context of cancer, in addition to autocrine ligand production, the stroma and/or basal lamina of vessels produce Netrins, thereby increasing the capacity of cells to migrate guided by NEO1. In cancer onset NEO1 might govern cell cycle kinetics and survival [6], while in aggressive tumor cells, NEO1 may function promoting cell migration. Different NTN4 availability might account for a differential behavior of NEO1 *in vivo* [36]. Further work is required to delineate the associated cellular mechanism required for cell survival and migration guided by NEO1 through ligand gradients, such as NTN4.

### *MYCN* amplification and NEO1 and NTN4 expression

As *MYCN* amplification is associated with NB aggressiveness and poor prognosis (MYCN protein expression as a predictor of neuroblastoma prognosis) [45], we evaluated the relation in between *MYCN* amplification and NEO1/NTN4 expression. According with Versteeg data set [23], there is not difference in *NEO1* expression in tumor samples with or without *MYCN* amplification (Supplementary Figure 4A). By contrast, *MYCN* amplified tumor samples have more *NTN4* expression (Supplementary Figure 4B), which is in line with the expression data observed in NB cell lines analyzed in this work. It is important to emphasize the complexity of the NEO1 signaling pathway, and how NTN4 acts through NEO1 regardless of *MYCN* status. Therefore, NEO1 could be one of the main regulators of survival and migration in NB.

On the other hand, there is controversy about *NTN1* expression in *MYCN* amplified tumors. In the same data set, its shown that *NTN1* is less expressed in *MYCN* amplified tumors (Supplementary Figure 4C), which does not correspond with cell lines analyzed in this work and with other literature, which shows that there is not an association between *NTN1* up-regulation and *MYCN* amplification [18]. NTN1 Kapplan Meier survival plots (Supplementary Figure 1B), show that NTN1 expression is a good survival prognosis factor, which is contrary with the *MYCN* amplification in NB aggressiveness. Importantly, previous report [18], have shown that NTN1 survival function is via UNC5H, and not through NEO1. This controversy shows that NTN1 regulation is complex and needs further analysis.

In conclusion, NTN4 and its receptor NEO1 promote cell migration, survival, and metastasis in NB cells. The NTN4/NEO1 signaling complex balances apoptosis and survival. If NTN4 is not expressed, NEO1 induces cellular apoptosis. However, if NTN4 is expressed, NEO1 signaling promotes cellular survival and migration. All these results underlie that there is a delicate balance between apoptosis and cell migration.

NEO1 signaling is becoming an attractive target for use in cancer therapies [6, 46]. Based on our results, we propose that NEO1 and/or NTN4 are promising targets for use anti-cancer therapies, in particular to inhibit the tumoral metastasis. Considering the ultimate efforts in the clinical and genomic medicine field, directed towards the generation of new therapeutic strategies, we could envision the development of inhibitors of the extracellular recognition of NTN4 by NEO1, allowing thus the modulation of NEO1 activity in an extracellular fashion.

## MATERIALS AND METHODS

### Cell culture

Neuroblastoma cell lines SH-SY5Y, SK-N-SH, LAN-1 and, NB1691 were cultured in high glucose Dulbecco's Modified Eagle Medium (DMEM, Invitrogen) with 5% (SK-N-SH) or 10% (LAN-1, NB1691) fetal bovine serum (FBS, Gibco) and supplemented with antibiotics (Penicillin-Streptomycin, 10,000 U/mL). The U87 (glioblastoma), HEK293 (human embryonic kidney) and DAOY (medulloblastoma) cell lines were cultured in DMEM with 10% FBS supplemented with antibiotics.

### Western blots

Protein extraction was realized using lysis buffer (SDS 2% w/v, Tris-HCL 80 mM pH 7.5, Glycine 10% w/v) with protease inhibitors (Thermo). After three minutes of sonication at ice-cold temperature, samples were centrifuged (10000 × g) for 5 minutes at 4°C. The antibodies used were anti-Neo1 (#sc-6536, Santa Cruz Biotechnology), anti-DCC (#sc-6535, Santa Cruz Biotechnology), anti-Netrin-1 (AF6419, R&D systems), anti-NTN4 (HYR01, R&D systems), anti-RGMa (AF2459, R&D systems), anti-actin (A5316, Sigma), and anti-tubulin (T9026, Sigma). Primary antibodies were incubated overnight at 4°C in 5% non-fat milk diluted in TBS-Tween 0,01% and secondary antibodies were incubated at room temperature for two hours in the same buffer. Western blots were quantified using integrated density analysis with ImageJ software (National Institutes of Health, USA).

### Protein co-immunoprecipitation

SK-N-SH cells were incubated with human recombinant Netrin-4 (200 ng/ml) for 1h. Later, cell extracts were prepared in a buffer containing 20 mM Tris, pH 7.4, 150 mM NaCl, 1% NP-40, and protease inhibitors by 5 min incubation on ice. Samples were centrifuged at 13,000 × g by 1 min at 4°C, and supernatants (1000 μg total protein) were immunoprecipitated with protein A/G bead-immobilized antibodies for 1h. NEO1 was immunoprecipitated with 5 μg of a rabbit polyclonal antibody (H-175 Santa Cruz) and NTN4 was immunoprecipitated with 5 μg of NTN4 goat polyclonal antibody. Immunoprecipitated samples were solubilized in loading buffer with ß-mercaptoethanol, and analyzed by Western blot as indicated in the Figure 1.

### Quantitative PCR

We used real-time quantitative PCR (qPCR) to identify and quantify the expression of *NTN4* in NB cells. The cell cultures were maintained in 10% FBS until 90% confluence, where they were used for RNA extraction using RNAsolv (OMEGA, R6830-02). Purified RNA was used for cDNA synthesis using Revertaid Reverse Transcriptase (Thermo scientific) according to the manufacturer's instructions. The primers used for qPCR analysis were: *NTN4* (Fw: 5′ TCAGCACAACACAGAAGGACA3′; Rv: 5′ GGATGGCAGGAACACGGTTTG 3′), and 40 PCR cycles were used in the experiments, at an annealing temperature of 60°C. Gene expression values were graphed as a fold change with respect to GAPDH (Fw: 5′ CAAGAAGGTGGTGAAGCAGGC3′; Rv: 5′CCACCACCCTGTTGCTGTAG3′). PCR amplification was verified via gel electrophoresis.

### Lentiviral transduction and stable shRNAs cell line generation

To knock-down *NEO1* (shNEO1) and *NTN4* (shNTN4), SK-N-SH and LAN-1 cells were transduced through lentiviral particles that contain shRNAs (pGIPZ backbone) vectors for each gene of interest [6]. A scramble sequence (shSCR) was used as a control. Lentiviral particles were prepared as indicated in [47]. Briefly, HEK 293T cells were triple transfected with pCMV-VSV-G, p8.91, and pGIPZ-shRNA (Openbiosystems). Viral supernatants were harvested 48 hours after transfection, filtered through 0.45-mm cellulose acetate filters, and them were used to transduce SK-N-H and LAN-1 cultured with this medium mixed with DMEM 5% FBS. After 48 hours, the transduction percentage was measured using tGFP encoding in pGIPZ and cells were incubated with the selection marker puromycin (3 μg/ml for SK-N-SH and 1μg/ml LAN-1, Sigma) for an additional 48 hours. Cells were maintained in DMEM with FBS supplemented with puromycin. The knock-down efficiency was measured via Western blot analysis.

### NEO1GFP and NEO1ICDGFP overexpression

The plasmids used to overexpress Neo1GFP and NeoICD (intracellular domain of NEO1) fragments were a kind donation from Dr. Patrick Mehlen (Université de Lyon, Centre Léon Bérard, Lyon, France), and were prepared as described in [48]. The backbone used for the plasmids was pEGP-C1, which has an eGFP sequence in frame with NEO1 or NEOICD sequence. The TrkC-overexpression plasmid was also a donation from Dr. Patrick Mehlen and the backbone used for the plasmid was pCDNA3, described in [49]. The transfection was made in SK-N-SH cells using Turbofect (Thermofisher) according to the manufacturer´s instructions.

### Wound healing assay

The wound healing assay was carried out using SK-N-SH shNEO1, shNTN4, and shSCR cells as described in [50], with some modifications. Briefly, cells were cultured for 24h to reach confluence. Then, a scratch was made with a micropipette tip in the center of the plate in order to generate a space between cells. The cells were washed with phosphate buffered saline (PBS) and incubated in DMEM 2.5% FBS. The plates were photographed using a microscope (Motic) coupled to digital camera (Leica) at 100x of total amplification, setting this at time 0. Cells were incubated for 9h and then photographed using the same conditions. This experiment was carried out in quadruplicate. The analysis was made using Image J software and the data are shown as a percentage of wound healing (closure) of each cell type.

### Transwell migration assays

Tranwell assays were completed using a chamber within an 8μm-pore polycarbonate membrane (Corning). As a haptotactic stimulus, 2μg/μl fibronectin was used (Sigma Aldrich), placed on the bottom of the membrane 12h before performing the assay. As a chemotactic stimulus, different concentrations of human recombinant Netrin-4 (R&D Systems) diluted in DMEM (without FBS) were used; the concentrations are indicated in Figure 4. Briefly, for SK-N-SH, 50,000 shNEO1, shNTN4, and shSCR cells were placed in the upper chamber; the bottom chamber contained NTN4 diluted in DMEM. The cells were incubated for 4h, fixed, and stained using Crystal violet 100% diluted in methanol in a solution 1:5 of NaCl 0,15M. For LAN-1 100,000 shNEO1, shNTN4, and shSCR cells were placed in the upper chamber; the bottom chamber contained 100 ng/ml Netrin-4 diluted in DMEM. The cells were incubated for 6h, fixed, and stained same as SK-N-SH cells.

To determine if NEO1-overexpressed cells migrate more in transwell assay than control cells in the presence of a Netrin-4 stimulus (100ng/mL), SK-N-SH cells overexpressing EV (empty vector), NEO1GFP, or NEO1ICDGFP were used. The results were normalized according to condition without Netrin-4 for each experiment.

### Immunofluorescence

Double-immunofluorescence were completed with anti-cleaved Caspase-3 (9661, Cell Signaling) and anti-Tubulin (T9026, Sigma) in SK-N-SH shNTN4 and shSCR cells. The cells were cultured in 24-well plates for 24h. Subsequently, they were deprived of FBS, and exogenous Netrin-4 was added for 24h. Human recombinant RGMa (100ng/mL) was used as a control. Cell nuclei were also stained with a DAPI (Sigma Aldrich).

To evaluate proliferation, SK-N-SH shNTN4, and shSCR cells were incubated with BrdU (Sigma) for 1h in 24-well plates in DMEM 2,5% FBS. Then, the cells were fixed with 4% paraformaldehyde (PFA) and immunostained with anti-BrdU (DAKO) and phosphohistone3 (Millpore) antibodies following the protocol as explained previously. Fluorescence microscopy was performed using an Olympus BX- 51 microscope. A minimum of three independent experiments was realized for each assay.

### Propidium iodide staining

LAN-1 cells shSCR and shNTN4 were culture in 96-well plates. After 24 h, cells were serum deprived by 24h. To evaluate dead cells, cells were stained with Propidium iodide (PI) and Hoechst at final concentration of 1 μg/ml and 5 μg/ml, respectively. Cells were analyzed and counted using Cytell Cell Imaging System (GE Healthcare), using Cell Viability BioApp (GEHealthcare). Ten fields were analyzed per well and average per well were grafted. The assay was made by six replicates for each condition and two independent experiments were realized for the assay.

### TUNEL assay

TUNEL assays were conducted utilizing the ApopTag® Fluorescein Direct *in Situ* Apoptosis Detection Kit (Merck Millipore), following the manufacturer's instructions, to measure apoptosis in shNTN4, shSCR, and NEO1-overexpressing SK-N-SH cells. Cells were incubated in Netrin-4 (200ng/mL) diluted in DMEM for 24h. We used DMEM without FBS as an incubation control. The assays were analyzed by fluorescence microscopy using an Olympus BX- 51 microscope. TUNEL positive cells and DAPI positive cells were counted and the ratio between both quantifications was graphed as a percentage.

### siRNA transfection

To evaluate the contribution of NEO1 in the apoptosis induced by NTN4 knock-down, we transfected siRNAs against NEO1 in shNTN4 SK-N-SH cells using Turbofect (Thermofisher) according to manufacture's instructions. The sequences correspond to siNEO1(1) (SASI_Hs02_00333957) and siNEO1(2) (SASI_Hs01_00151269) and, siControl (SIC002) provided by Sigma Aldrich. siRNA efficiency was evaluated through Western blot against NEO1. Briefly, 1μg of siRNA was transfected into shNTN4 cells and 48 h later, cells were serum deprived for 48h and fixed with PFA 4%. Apoptosis was evaluated via TUNEL and immunofluorescence of Cleaved-Caspase-3. The assays were analyzed by fluorescence microscopy using an Olympus BX- 51 microscope. Positive cells and DAPI positive cells were counted and the ratio between both quantifications was graphed as a percentage. The assays were made by 30 replicates for each condition and two independent experiments were realized for each assay.

### Metastasis analysis via CAM assays

Fertilized chicken eggs were incubated at 37.5°C with constant humidity. On the second day of incubation (E2), 2 mL of albumin was removed from the egg. On day four (E4), a rounded window was made in the shell in order to have access to the chick chorioallantoic membrane (CAM), and sealed with adhesive tape. On day ten of incubation (E10), ten million SK-N-SH shNEO1, shNTN4, or shSCR cells were drop-plated on the developing CAM. On day 17 of incubation (E17), the primary tumor of the CAM was weighed and the embryo was dissected. Embryonic lungs were incubated with RNASolv (OmegaBiotek) and kept at -20°C. We extracted genomic DNA following the manufacturer's instructions. Genomic DNA expression levels were analyzed via qPCR analysis using human *Alu* sequences (FW: 5′ACG CCT GTA ATC CCA GCA CTT3′; RV: 5′TCG CCC AGG CTG GAG TGC A3′) and genomic chicken GAPDH (FW: 5′GAG GAA AGG TCG CCT GG3′; RV: 5′GGT GAG GAC AAG CAG TGA3′) primers. The analysis was made using fold change with respect to chGAPDH and normalized according to lung of control cells (shSCR). Five eggs were used for each condition.

## SUPPLEMENTARY FIGURES


