# Supplementary material for: The Netrin-4/ Neogenin-1 axis promotes neuroblastoma cell survival and migration
Source: Oncotarget. 2016 Dec 25;8(6):9767–82. doi: 10.18632/oncotarget.14213 (PMC5354769; doi:10.18632/oncotarget.14213)
Supplement: Supplementary file 1 [file oncotarget-08-9767-s001.pdf]

The Netrin-4/ Neogenin-1 axis promotes neuroblastoma cell survival and migration

SUPPLEMENTARY FIGURES

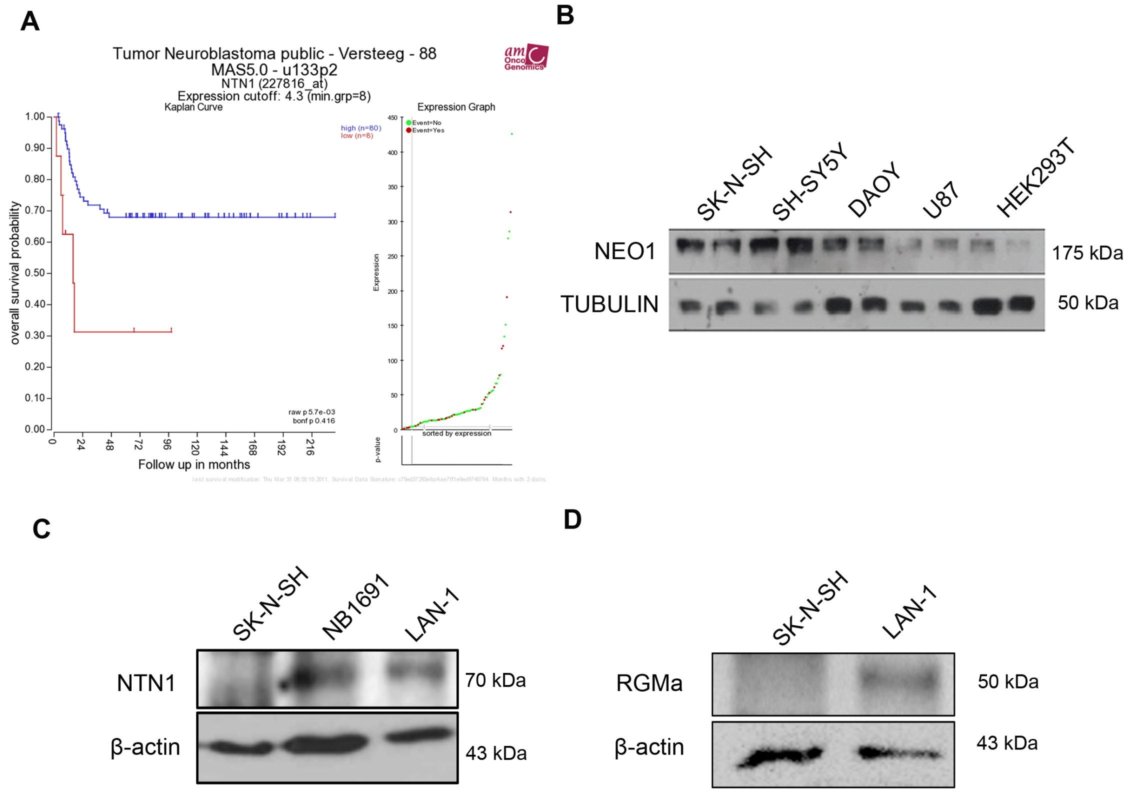

Supplementary Figure 1: Characterization of NEO1 and its ligands NTN1 and RGMa in tumor cell lines. A. Clinical significance of NTN1 expression in NB. *NTN1* mRNA value is plotted against patient survival rate in a Kaplan-Meier estimate plot. **B.** Western blot against NEO1in several tumor cell lines, as indicated. **C.** Western blot of NTN1 in both WT and high *MYCN* NB cell lines. Note that NTN1 is not expressed in SK-N-SH cells. **D.** Western blot against RGMa in representative WT and high *MYCN* NB cell lines. Note that RGMa is not expressed in SK-N-SH cells.

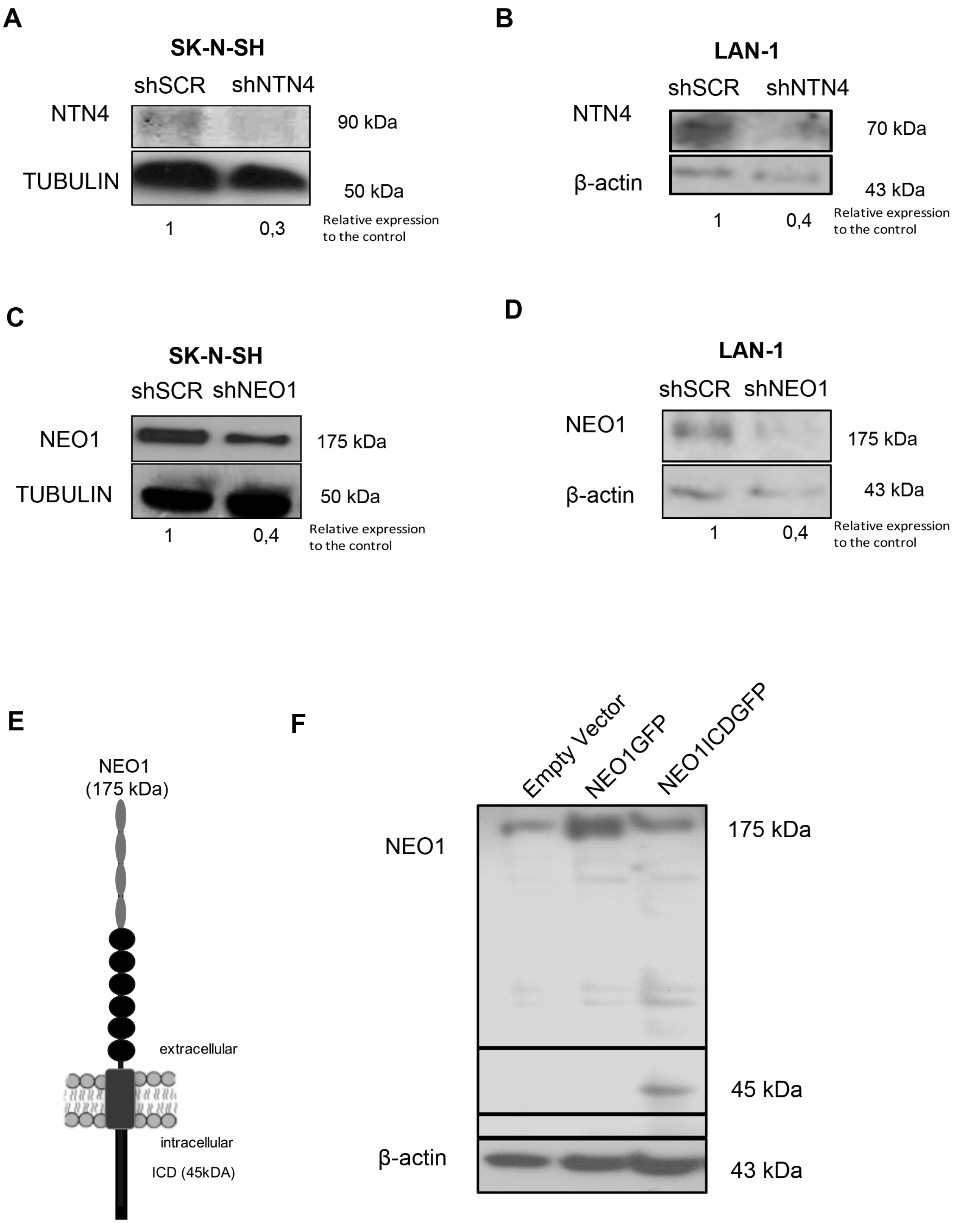

Supplementary Figure 2: NEO1 and NTN4 knock-down and NEO1 overexpression in NB cell lines. A, B. Representative Western blots against NTN4 in stable transduced cells using lentiviral particles containing shRNA for NTN4 or shSCR control in SK-N-SH (A) and LAN-1 (B). Relative expression to the control value was estimated from a triplicate. **C, D.** Representative Western blots against NEO1 in stable transduced cells using lentiviral particles containing shRNA for either NEO1 or shSCR control in SK-N-SH (C) and LAN-1 (D) cells. Relative expression to the control value was estimated from a triplicate. **E.** Schematic representation of NEO1 depicting the structure of the full- length protein and the intracellular NEO1 domain (ICD). **F.** Western blot against NEO1 revealing its expression in cells transfected with an empty vector, NEO1 (NEO1GFP), and NEOICD (NEO1ICDGFP). Note that the 45kDa band is only detected when overexpressing NEOICDGFP (see box).

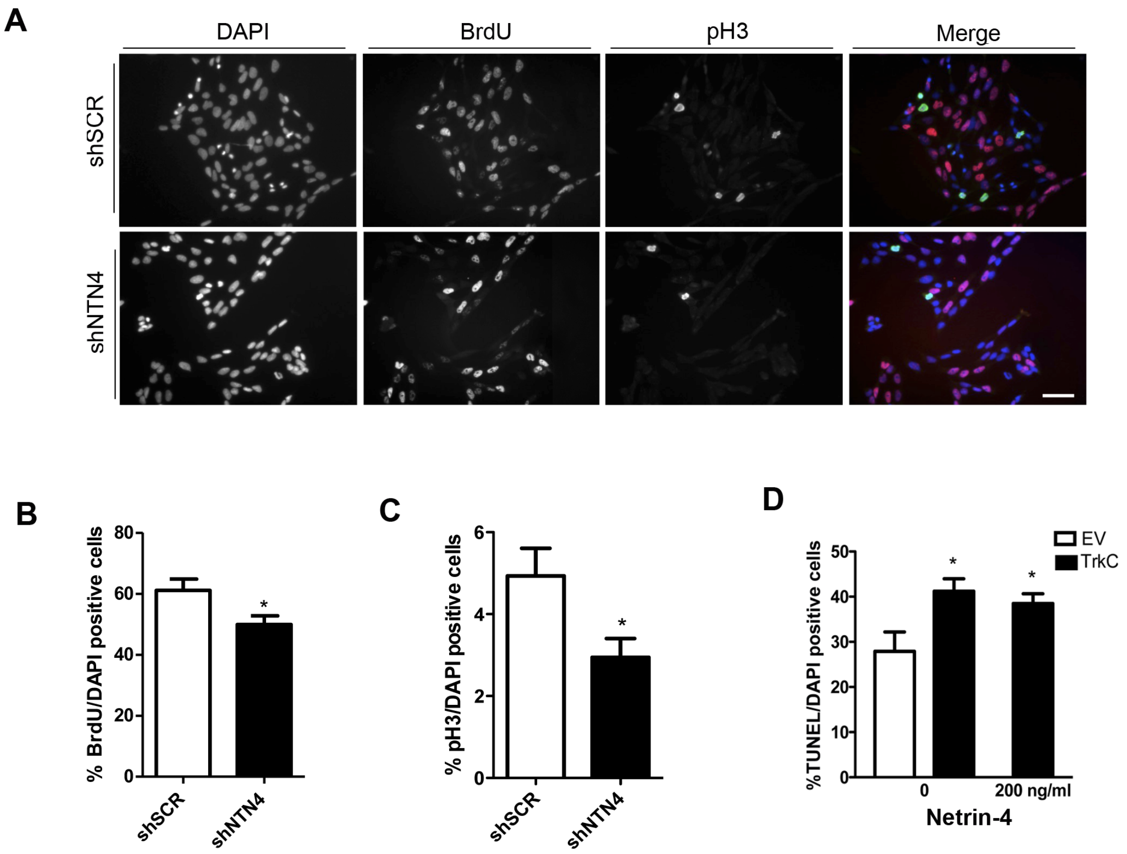

Supplementary Figure 3: NTN4 knock-down reduces the proliferation in SK-N-SH cells and Netrin-4 treatment cannot reduce apoptosis induced by TrkC in SK-N-SH cells. A. Representative images of immunofluorescence against phospho-Histone-3 (pH3) and BrdU. Bar:100 µm **B, C.** Quantification of BrdU (B) and pH3 (C) positive cells in shSCR or shNTN4. *p<0,05. **D.** TUNEL assay of SK-N-SH cells transfected with TrkC or empty vector (EV) and treated in serum free media for 24 h with or without Netrin-4 (200 ng/ml) EV vs TrkC*p<0,05.

A

NEO1

B

NTN4

C

NTN1

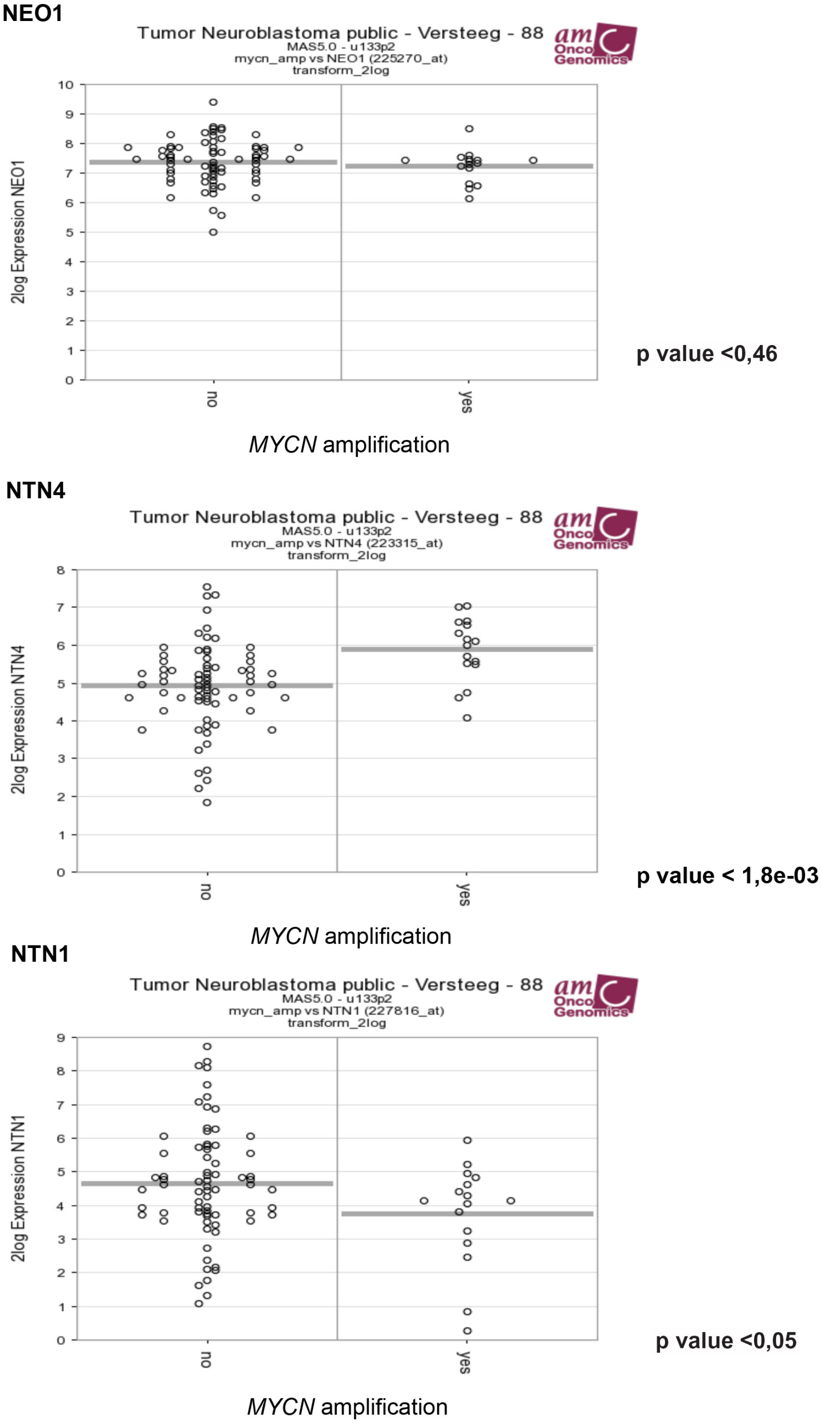

Supplementary Figure 4: *NEO1* and *NTN* expression according to *MYCN* amplification. A-C. Using the public primary tumor NB database from 88 patients (Versteeg data set), *NEO1* (A), *NTN4* (B) and *NTN1* (C) expression was plotted according to *MYCN* amplification in patient samples and expressed as yes (*MYCN* amplification) or no (*MYCN* WT). p value is indicated in the figure for each gene.
